# Diagnostic Utility of Cytology in Assessment of Ploidy Status in Potentially Malignant Oral Disorders

**DOI:** 10.31557/APJCP.2019.20.10.3145

**Published:** 2019

**Authors:** Thayalan Dineshkumar, Prabakar Srikanth, A E Nagarathinam, Krishnan Rajkumar, Shankaran Priyadharini, T A Shruthi

**Affiliations:** *Department of Oral and Maxillofacial Pathology, SRM Dental College and Hospitals, SRM Institute of Science and Technology, Ramapuram, Chennai, India. *

**Keywords:** Cytology, ploidy, PMODs

## Abstract

**Introduction:**

Oral leukoplakia, the most common potentially malignant oral disorder (PMOD) may progress to oral squamous cell carcinoma (OSCC). Although, the current standard of care for assessing its malignant potential remains histological examination and assessing the severity of dysplasia, DNA ploidy analysis has been suggested as a surrogate marker to predict the behaviour of PMODs.

**Objectives:**

To detect aneuploidy and to correlate ploidy status with different grades of dysplasia in both tissue and cytology samples to predict the behaviour of these potentially malignant disorders and to assess the diagnostic utility of cytology samples for ploidy analysis.

**Methodology:**

After obtaining ethical clearance and consent, tissue and cytology samples of leukoplakia were collected and grouped based on the dysplastic findings into low-risk (n=20) and high-risk (n=20). DNA ploidy analysis was done using high resolution flow cytometry and its diagnostic utility was assessed.

**Results:**

Diagnostic utility was expressed in terms of sensitivity, specificity, PPV and NPV. On comparing the ploidy status of individual cases between tissue and cytology samples, cytology was able to accurately determine the ploidy status in majority of the cases. In the low-risk group, cytology had a sensitivity and specificity of 100% and a PPV and NPV of 100% with an overall diagnostic accuracy of 100%. Among the high-risk group, cytology had a sensitivity of 80% and specificity of 100% with a PPV of 100% and NPV of 83.33% and had an overall diagnostic accuracy of 90%. Combining both groups together, it had a sensitivity of 85.71% and specificity of 100% with a PPV of 100% and NPV of 92.31% and had an overall diagnostic accuracy of 94.74%.

**Conclusion:**

Overall, this study showed a positive correlation between cytology and tissue samples and ploidy and grade of dysplasia and cytology proved to be a simple and efficient with a reasonable diagnostic value.

## Introduction

Oral Squamous Cell Carcinoma (OSCC) is a multifactorial disease, where numerous intrinsic and extrinsic factors play a role in its causation. Regardless of the accelerating factors, OSCC arises from clonally transformed cells that have undergone specific genetic and epigenetic alterations in oncogenes or tumor suppressor genes (Bsoul et al., 2005).

As a result of these genotoxic insults from various etiological factors, the normal epithelia may undergo morphological changes with time, leading to appearance of lesions which have the potential to turn into malignancy and are termed as potentially malignant oral disorders (PMODs) (Castagnola et al., 2011; Mithani et al., 2007). There are several histologically distinct lesions of the oral mucosa which are characterized as having malignant potential, the majority of which present as leukoplakia. 

Leukoplakia is defined as ‘a white plaque of questionable risk having excluded other known disorders that carry no increased risk for cancer’ (Warnakulasuriya et al., 2007). The estimated prevalence of oral leukoplakia worldwide is approximately 2%, with an annual malignant transformation rate into OSCC of approximately 1% (Petti et al., 2003; Van der Waal et al., 2009). However, the prevalence of leukoplakia in India varies from 0.2% to 5.2% and the malignant transformation rates varied from 0.13% to 10% in various Indian populations (Mehta et al., 1969; Singh et al., 2004). 

Several factors are known to be associated with an increased risk of malignant transformation of leukoplakia. Currently, the presence of epithelial dysplasia is the gold standard in evaluating the malignant potential of suspicious lesions of the oral mucosa (Dietrich et al., 2004; Holmstrup et al., 2006; Warnakulasuriya et al., 2008). Unfortunately, these predictors are subjective, often lack sensitivity and hence are relatively unreliable, as often two lesions with identical grading behave in totally different fashions. 

On the basis of these large differences in reliably predicting the behaviour of these lesions, the management of PMODs becomes controversial. However, it is a known fact that before any clinical changes can occur in the offended mucosa, genetic damage occurs. Hence, it is logical to assume that genetic based studies can offer a better understanding of the biologic behaviour of PMODs. Recent studies have reported the potential use of DNA ploidy analysis to predict the behaviour of OSCC and oral PMODs (Khanna et al., 2010).

As a surrogate to individual molecular markers such as mutations in p53, loss of heterozygosity and chromosomal polysomy, measurement of gross genomic damage in the form of aberrant DNA content could be a valuable method for prognostication of malignant and premalignant lesions (Riháková et al., 2001). If any correlation between DNA ploidy and the histological grade of dysplasia can be demonstrated, it might be used as an adjunctive aid for pathologists to arrive at a consensus in diagnosing the grade of epithelial dysplasia. 

Although, recent studies have reported the potential use of DNA ploidy analysis to predict the behaviour of PMODs and also have shown that aneuploidy in oral dysplastic lesions, to be indicative of a high risk of OSCC development, these methods utilized DNA image flow cytometry which is less sensitive in detecting ploidy status. 

High-resolution flow cytometers are equipped with UV laser for fluorochrome excitation allowing DAPI (406-diamidino-2-phenylindoledihydrochloride) to be used for DNA staining. This allows for a lower coefficient of variance (CV) to be obtained to enable the detection of peridiploid-aneuploidy cell populations that would have been reported as diploid with less sensitive techniques.

Also, though histology is the gold standard for diagnosis and grading of PMODs, screening by taking random biopsies of both clinically normal and suspected oral tissue is not practical, since this causes serious discomfort to the patient and is not suited for repeated sampling at multiple sites. Alternatively, cytology has been proved to be a non-invasive, inexpensive and yet an established diagnostic tool. Hence, this study is performed by taking advantage of high resolution flow cytometry (FCM) to detect aneuploidy and to correlate ploidy status with different grades of dysplasia in both tissue and cytology samples to predict the behaviour of these potentially malignant disorders and to assess the diagnostic utility of cytology samples for ploidy analysis. 

## Materials and Methods

*Study Groups*

The study was approved by the ethical committee of SRM University. The details of the study were explained to all patients and written and informed consent was obtained from all patients before enrolling into the study. Patients for the study were recruited from the Outpatient Department of SRM Dental College and Referrals from Private Dental Clinics. 

G-power 3.1 software was used for sample size determination. Calculated sample size based on the effect size (d=1.44, derived from previously conducted study), α error = 0.05 and power = 0.95 was 12 in each group. 20% sampling errors were expected in each group and a final sample size of 20 in each group was arrived at. 

The study included a total of 40 patients (29 males and 11 females) belonging to age group of 23-61 years. Patients with clinically diagnosed potentially malignant lesions (leukoplakia) were included in the study. Patients with recurrent premalignant lesions, currently undergoing or having undergone any form of treatment for oral leukoplakia and those with excessive ulceration and extensive inflammation were excluded.

Grading of dysplasia was done by binary system based on World Health Organization grading criteria of dysplasia 2005 as low-risk and high-risk. In order to eliminate the observer bias, the samples were doubleblinded and assessed by two oral pathologists for grading of dysplasia.

The selected patients were grouped into either low-risk or high-risk group based on histological grading.

Group 1. Low risk (former “no/ mild / questionable” dysplasia) – 20 cases

Group II. High risk (former “moderate / severe” dysplasia) – 20 cases

*Sample Collection*

Incisional biopsy was performed at identical sites in patients, clinically diagnosed as Oral Leukoplakia. After biopsy, the tissue samples were cut into two half’s and one half was fixed in 10% formalin and routine histopathological processing and staining was performed and histopathological diagnosis was made. The other section of tissue was transferred to the cytometry lab in an ice bag at –4°C and was processed immediately for determining the ploidy status.

Oral mucosal keratinocytes were harvested using Oral Cytobrush (“Touchless” One-slide PAP smear kit) from patients clinically diagnosed as Oral Leukoplakia. Each patient was asked to rinse their mouth with phosphate buffered saline (PBS - pH 7.4) at least once to remove loose surface cells and to reduce the levels of bacterial content in the samples. The initial brushing was also discarded because of the presence of superficial layer of oral keratinocytes which was found to be more than 70% apoptotic populations. Cells from the identical lesional site were brushed at least 10 times. After each brushing the cells were immediately placed in 15ml falcon tube containing PBS (pH7.4) and transferred for ploidy analysis.

*Processing of samples for Flow cytometry analysis*

For tissue preparation, a 25-gauge x 1.5-inch needle tip was introduced into the tissue sample and once positioned, the syringe plunger was pulled back gently in order to produce a vacuum. Once a visible amount of material appeared in the needle hub, the vacuum was released and the needle was withdrawn from the tissue. The aspirated cells were carefully expelled into Eppendorf tubes containing 1mL of PBS. The PBS solution was aspirated into the hub (not into the syringe) and this wash PBS was again expelled into the same sample tube. The above steps were repeated until sufficient amount of cells (5.0 x 10^5 ^cells) were collected in the PBS suspension, which was confirmed by cell counting using haemocytometer. 

For preparation of Oral Keratinocytes (Cytology samples), the cell suspension was centrifuged at 300 rpm at room temperature for 5 minutes. The supernatant was aspirated leaving approximately 50µL of the residual fluid in the tube to avoid disturbing the pellet. The residual cells were again suspended in 10mL of PBS. Cell counting was done by standard laboratory methods using haemocytometer. 

For both the samples, suspensions of DNA were prepared using Cycle test plus DNA reagent Kit. (Cat. No. 340242) (BD bioscience) and staining for FCM measurement was carried out by exposure to 10 μg/ml DAPI. The amount of DNA was measured by BD FACS Jazz (BD Biosciences, Sanjose, California). FCM was performed on nuclei obtained from the specimens. DNA histograms of at least 10,000 cells were plotted. The diploid cell populations were used as an internal reference standard for the identification of aneuploid clones. Cell cycle analysis was done using Guava cell cycle pro software.

Statistical analysis of the data obtained was done using SPSS software. The association between ploidy status and the histologic grade of dysplasia was evaluated with the Chi-square test. A p-value of <0.05 was considered statistically significant. The diagnostic utility of cytology was evaluated in terms of sensitivity, specificity, positive predictive value (PPV) and negative predictive value (NPV).

## Results

Demographic and clinical characteristic of the patients are outlined in Table 1. Briefly age and sex matched 40 subjects who were clinically diagnosed as oral leukoplakia were included in the study, and categorized into low-risk and high-risk based on histologic grading and comprised of 20 subjects in each group. Grading was done and the overall distribution based on the histopathological grading of leukoplakia included 2 subjects with Hyperorthokeratosis without dysplasia, 18 subjects with mild dysplasia, 14 subjects with moderate dysplasia and 6 subjects with severe dysplasia. 

**Figure 1:**
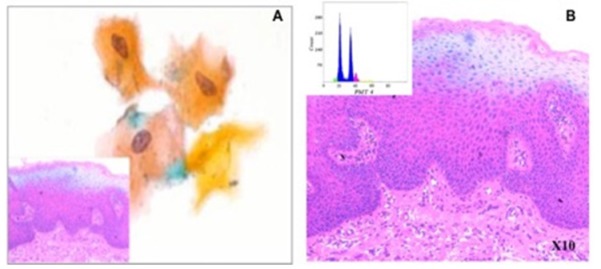
A, Cytological findings show mild dysplastic features: enlarged nuclei with slight hyperchromasia and orange cytoplasm. (Pap x400) [Inset: corresponding histopathology image]; B, Ploidy analysis of the same case demonstrated an aneuploid DNA histogram (inset)

**Figure 2:**
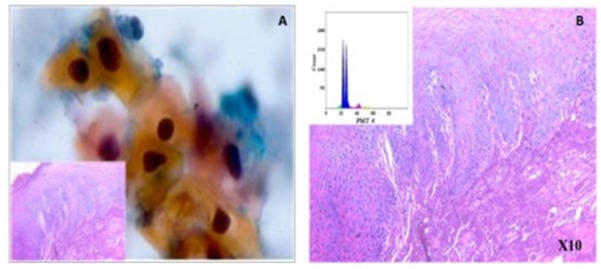
A, Cytological findings show severe dysplastic changes: nuclear hyperchromasia, irregular border and thick keratinized cytoplasm (Pap x400) [Inset: corresponding histopathology image]; B, Ploidy analysis of the same case demonstrated an aneuploid DNA histogram (inset)

In tissue samples, aneuploidy was found in 20% of low grade and 50% of high grade dysplasia’s (Figure 1,2), which showed a clear correlation between histologic grade of the leukoplakias and DNA ploidy status and these differences were statistically significant (p = 0.046) (Table 2). Aneuploidy in cytology samples, was found in 20% of low grade and in 40% of high grade dysplasias, which showed a positive correlation between the grade and ploidy status, although not statistically significant (p = 0.239) (Table 2). However, in low grade, 2 cases (10%) showed apoptotic cells.

**Table 1:** Demographics and Clinical Characters of Leukoplakia

Category	Low Risk (n = 20)		High Risk (n = 20)	
	Hyperorthokeratosis without dysplasia	Mild dysplasia	Moderate dysplasia	Severe Dysplasia
Subjects	2	18	14	6
Age range (years)	35 – 42	23 - 58	24 - 61	28 - 57
Sex				
Male	1	13	11	4
Female	1	5	3	2
Habits				
Tobacco chewing	2	8	5	2
Smoking	-	2	3	-
Smoking and chewing	-	5	4	4
Smoking and alcohol	-	3	2	-
Clinical type of leukoplakia				
Homogenous	2	14	6	-
Non-homogenous	-	4	8	6
Size of lesion				
<2 cm	2	10	1	-
2-4 cm	-	6	5	1
>4 cm	-	2	8	5
Site				
Buccal Mucosa	2	13	6	2
Buccal mucosa & vestibule	-	4	5	3
Tongue	-	1	1	1
Lip	-	-	2	-

**Table 2:** Ploidy Status of Samples in Different Degrees of Dysplasia

Sample	Groups	Diploid n (%)	Aneuploid n (%)	Apoptotic n (%)	p value
Tissue	Low grade (n=20)	16 (80%)	4 (20%)	Nil	0.046
	High grade (n=20)	10 (50%)	10 (50%)	Nil	
Cytology	Low grade (n=20)	14 (70%)	4 (20%)	2 (10%)	0.239
	High grade (n=20)	12 (60%)	8 (40%)	Nil	

p-value determined by Chi square test; p-value of less than 0.05 was considered significant

**Table 3:** Diagnostic Utility of Cytology in Determining Ploidy Status

Diagnostic Parameter	Low Risk	High Risk	Combined (Low & High Risk)
Sensitivity	100%	80%	85.71%
Specificity	100%	100%	100%
Positive Predictive Value (PPV)	100%	100%	100%
Negative Predictive Value (NPV)	100%	88.33%	92.31%
Area under Curve	1.000	0.7229	0.833

Diagnostic utility assessed in terms of sensitivity, specificity, positive predictive value, negative predictive value

**Figure 3:**
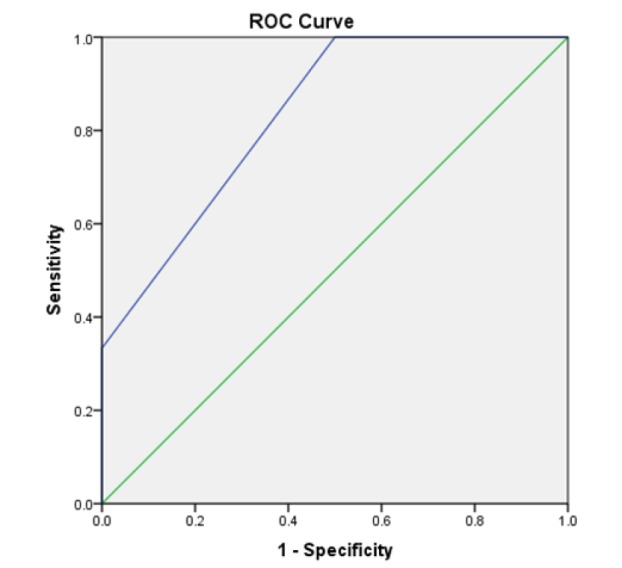
Diagnostic Utility of Cytology Assessed by ROC Curve Analysis. ROC curve analysis of cytology (combined low-risk and high-risk) for predicting ploidy when compared with biopsy

Diagnostic utility of cytology was expressed in terms of sensitivity, specificity, PPV and NPV. On comparing the ploidy status of individual cases between tissue and cytology samples, cytology was able to accurately determine the ploidy status in majority of the cases. In the low low risk group, cytology had a sensitivity and specificity of 100% and a PPV and NPV of 100% with an overall diagnostic accuracy of 100%. Among the high risk group, cytology had a sensitivity of 80% and specificity of 100% with a PPV of 100% and NPV of 83.33% and had an overall diagnostic accuracy of 90%. Combining both groups (low risk and high risk) together, it had a sensitivity of 85.71% and specificity of 100% with a PPV of 100% and NPV of 92.31% and had an overall diagnostic accuracy of 94.74% (Table 3). Curve comparing the diagnostic ability of cytology in detecting ploidy status using ROC was done and AUC for low-risk cases is 1.000 and high-risk cases 0.91 and combined for both groups is 0.96 (Table 3 and Figure 3).

## Discussion

OSCC is often preceded by the appearance of lesions which have the potential to develop into invasive carcinoma and theoretically, identification and elimination of cancer precursors would lead to the eradication of most human cancers, suggesting that precancers are the most important lesions in modern man (Jules, 2003).

Caner is considered to be a two-step process consisting of an initial precursor lesion which is potentially malignant, subsequently developing into cancer. Of the many known precursor lesions, oral leukoplakia is the most common potentially malignant lesion of the oral cavity. At present, histopathological assessment and identifying the presence of epithelial dysplasia is the gold standard for the prediction of malignant transformation of potentially malignant disorders. Hence, the need for regular monitoring of these common potentially malignant lesions is of paramount importance as the risk of malignant transformation of leukoplakia with dysplasia has been reported to be high (Reibel et al., 2003; VázquezÁlvarez et al., 2010). 

However, the presence of epithelial dysplasia as a predictor in malignant transformation in these lesions is still controversial as not all lesions exhibiting dysplasia will eventually become malignant and carcinoma can develop from lesions in which epithelial dysplasia was not present in biopsy specimens (Schepman et al., 1998; Pindborg et al., 1977; Silverman Jr et al., 1984). 

Such contradictory reports and lack of information in the literature to reliably predict the biological behaviour of these lesions are the main reasons why the management of PMOD is still controversial. As a consequence of these problems, numerous attempts have been made to relate biological characteristics to the malignant potential of leukoplakia. It is believed that ploidy analysis is important in prediction of carcinogenesis. DNA aneuploidy can be defined as any imbalance of chromosomal material and any amplification or deletion of DNA influences the expression of a multitude of genes, regardless of whether such changes occur in chromosomal structure or number. Ploidy studies on other cancer types have shown DNA aneuploid lesions in Barrett’s esophagus to have a higher risk of malignant transformation (Fang et al., 2004) and DNA aneuploid gastric cancer has an unfavourable prognosis compared to DNA diploid cancer (Belien et al., 2009).

It has been shown that in some PMODs the epithelial cells exhibit changes from a diploid pattern to an aneuploid pattern preceding malignant transformation, which means that DNA alterations take place before transformation is apparent and is seen as a surrogate marker of gross genetic damage. 

Although, histology remains the gold standard for diagnosis and grading of oral carcinomas and PMOD lesions, screening by taking random biopsies of both clinically normal and suspected oral tissue is not practical, since this causes serious discomfort to the patient and is not suited for repeated sampling at multiple sites. Alternatively, cytology has been proved to be a non-invasive, inexpensive and yet an established diagnostic tool.

In the present study aneuploidy was found in 20% of low grade and 50% of high grade dysplasia’s, which showed a clear correlation between histologic grade of the leukoplakias and DNA ploidy status. This was in accordance with Saito et al 1991, who analysed the DNA ploidy status in 19 oral leukoplakias with and without epithelial dysplasia using flow cytometry and compared with 11 normal gingival biopsies. The results showed that DNA aneuploidy clones in 32% of the oral leukoplakia, while all the normal gingival biopsies showed diploid clones (Saito et al., 1991). 

Sudbø et al., (2001), also showed a positive relation between ploidy and dysplasia and also showed that the frequency of carcinomatous transformation of dysplastic oral leukoplakia is much greater for those leukoplakias with keratinocytes showing DNA aneuploidy than for those with keratinocytes with normal (diploid) DNA content, and subjects with oral carcinoma developing from dysplastic leukoplakia with keratinocytes manifesting aneuploidy have a lower rate of survival compared to subjects with oral squamous cell carcinoma evolving from dysplastic leukoplakia with keratinocytes with diploid DNA content. 

Brouns et al., (2012), demonstrated that DNA aneuploid lesions were more frequently encountered at high-risk locations as being determined with flow cytometry. However, these relations were not found when DNA ploidy was determined with image cytometry proving that flow cytometry was more sensitive in detecting DNA changes.

Andre et al., (2012), also found significant correlation between the degree of dysplasia and ploidy status. Also, when they grouped the dysplasia into low-risk and high-risk categories the differences in ploidy status were more prominent and proposed that the binary grading system improved the well-documented inter-observer disagreement regarding grading of epithelial dysplasia.

Similar correlation was also found in a study by Vijayavel et al., (2013) who found statistically significant correlation between histopathology and ploidy in both potentially malignant and malignant group and interpreted that DNA ploidy analysis can be used as a valuable tool in assessing the carcinomatous progression of potential malignant lesions.

Contradicting results were reported by Torres-Rendon et al., (2009) and Bremmer et al., (2011) who found no correlation between histologic grade and DNA aneuploidy. However, the aneuploid dysplastic lesions in their study had a significantly increased risk to progress to OSCC compared to diploid dysplastic lesions (Torres-Rendon et al., 2009; Bremmer et al., 2011).

There are other contradicting reports in the literature concerning the correlations of DNA ploidy with grading. These discrepancies can be explained by the fact that the majority of these studies used image cytometry and different grading systems and scores that have been applied. Because FCM results are based on large numbers of nuclei measured for statistical analysis, distinct stem lines could be further resolved and more precise determination of DNA index values are possible, which makes FCM more reliable. 

Although, number of tissue based FCM techniques are used to study the genetic and molecular abnormalities of oral keratinocytes in premalignant and malignant oral lesions, to the best of our knowledge only limited studies exist on the validation of oral cytology to assess risk for premalignant lesions.

Aneuploidy in cytology samples, was found in 20% of low grade and in 40% of high grade dysplasias which showed a positive correlation between the grade and ploidy status. On comparing the ploidy status of individual case between tissue and samples, cytology was able to accurately determine the ploidy status in majority of the cases. The diagnostic utility of cytology in accurately determining the ploidy samples was determined in terms of sensitivity, specificity, positive predictive value (PPV) and negative predictive value (NPV).

Among the low risk group, cytology had a sensitivity and specificity of 100% and a PPV and NPV of 100% with an overall diagnostic accuracy of 100%. Among the high risk group, cytology had a sensitivity of 80% and specificity of 100% with a PPV of 100% and NPV of 83.33% and had an overall diagnostic accuracy of 90%. Combining both groups (low risk & high risk) together, it had a sensitivity of 85.71% and specificity of 100% with a PPV of 100% and NPV of 92.31% and had an overall diagnostic accuracy of 94.74%.

However, it has to be emphasized at this point that sampling errors could happen in cytology as we encountered in this study. Ideally, each patient has to first rinse with phosphate buffered saline (PBS) once to remove loose surface cells and decrease the level of bacteria. The initial brushing with the cytobrush has to be discarded because it contained primarily the superficial stratum corneum oral keratinocytes which was found to be >70% apoptotic which is not suitable for ploidy analysis.

In two of the cytology samples, subgenomic clones were found in abundance due to collection of superficial apoptotic cells. This cytology sample was not included in the statistical analysis. We purposefully included that sample in the study, to emphasize that cytology though a simple technique; caution should be exercised in sampling.

Overall, this study showed a positive correlation between cytology and tissue samples and ploidy and grade of dysplasia and cytology proved to be a simple and efficient method with a reasonable diagnostic value. 

*Limitations*

Although a positive correlation was shown, the study is not without its own limitations. It was beyond the current scope of this study to do a prospective follow up of those patients who demonstrated aneuploid status and hence there is no confirmatory evidence to deduce the malignant transformation status of these cases. Hence to determine the prognostic value of DNA ploidy status, it would be ideal when the clinical endpoint of development of OSCC is observed. 

In conclusion, the results of the present study demonstrated a strong association between DNA ploidy status and the presence of epithelial dysplasia. Hence DNA aneuploid leukoplakia lesions have a significant higher risk for OSCC progression, although DNA diploid lesions are not exempt from malignant progression. Unfortunately however, there were not enough patients with progression to cancer to determine the possible relation between DNA ploidy and malignant transformation.

But the demonstrated association between DNA ploidy status and the presence of epithelial dysplasia suggests that regular follow-up for the early detection of any possible mucosal alteration should be the minimum standard of care.

It must be emphasised that PMOD should only be regarded as indicator lesions reflecting the status of the oral mucosa. It is furthermore wrong to assume that OSCC will always develop from a pre-existing PMOD. This concept is in all likelihood one of the reasons why removal of PMOD has not significantly influenced the development of OSCC over the last few decades. Although there are uncertainties regarding the most appropriate treatment of patients with PMOD, DNA ploidy determination by high-resolution flow cytometry should be considered as a marker to predict biological behaviour in PMOD and could be an additional tool in determining the prognosis of oral dysplastic lesions.
